# The Future of Community Pharmacy Practice in South Africa in the Light of the Proposed New Qualification for Pharmacists: Implications and Challenges

**DOI:** 10.5539/gjhs.v6n6p226

**Published:** 2014-08-15

**Authors:** Ntambwe Malangu

**Affiliations:** 1 School of Public Health, University of Limpopo, Medunsa Campus Pretoria, South Africa

**Keywords:** pharmacists prescribing, qualification, South Africa

## Abstract

**Objectives:**

Community or retail pharmacies are regarded as one of the most common sources of health services throughout the world. In South Africa, community pharmacies have been providing some primary health care services to clients who could afford to pay. These services included screening, family planning, and emergency care for minor ailments. With the introduction of the new qualification, community pharmacies are poised to become providers of expanded services. This paper describes the contents, the implications and challenges of the new qualification in light with future directions for community pharmacy practice in South Africa. Its purpose is to inform relevant stakeholders in South Africa and those outside South Africa that may pursue similar offerings.

**Methods:**

Published papers were identified through searches in MEDLINE and Google Scholar using a combination of search terms, namely: ‘community, retail pharmacy, pharmacist/non-medical prescribing, South Africa’. Only articles published in English were considered. In addition, documents from the Ministry of Health of South Africa, the South African Pharmacy Council and curricula materials from schools of pharmacy were also reviewed.

**Key Findings:**

Laureates of the new qualification will essentially have the right to examine, diagnose, prescribe and monitor the treatment of their clients or patients. In doing so, this expanded function of prescribing for primary healthcare will imply several practice and infrastructural adjustments; and with many challenges laying ahead in need to be addressed.

**Conclusions:**

In conclusion, the authorized pharmacist prescriber qualification augurs a new era for community pharmacy practice in South Africa. This has many implications and some challenges that need to be managed. The pharmacy profession, academia, legislators and political decision-makers need to work together to resolve outstanding issues in a constructive manner.

## 1. Introduction

Community or retail pharmacies are regarded as one of the most common sources of health services throughout the world. In South Africa, community pharmacies have been providing some primary health care services to clients who could afford to pay for them. These services included screening tests, family planning and emergency care for minor ailments (Goel et al., 1996; Adepu & Nagavi, 2003; Dugan, 2006; Smith, 2004; Sello et al., 2012). Although clinically oriented, these services do not meet the needs of patients that frequent pharmacy. Apart for supply chain management skills, pharmacists are fundamentally trained to apply professional judgment in the provision of their services. However, regulatory measures have been established to prevent pharmacists from supplying or prescribing most common medicines from Schedule 3 of the South African Medicines Act 101 of 1965. To do so, pharmacists were required to do additional training to be certified for this. However, due to limited incentives, few pharmacists pursued the additional training. With the introduction of the new qualification, community pharmacies are poised to become providers of expanded services. In this paper, the contents, the implications and challenges of the new qualification are discussed with regard to the future directions of community pharmacy practice in South Africa. Its purpose is to inform relevant stakeholders in South Africa and those outside South Africa that may pursue similar offerings. It is not intended to provide an analysis of the merits or demerits of the qualification.

## 2. Methods

Published papers were identified through searches in MEDLINE and Google Scholar using a combination of search terms, namely: ‘community, retail pharmacy, pharmacist/non-medical prescribing, South Africa’. Only articles published in English were considered. In addition, documents from the Ministry of Health of South Africa, the South African Pharmacy Council and curricula materials from schools of pharmacy were also reviewed.

## 3. Results

Our initial screen resulted in 210 peer-reviewed publications from MEDLINE and Google Scholar. Less than ten papers were about South Africa. Of these, the majority of these papers were authored by one author, Leah Gilbert (Gilbert, 1998a, 1998b, 1998c, 2001, 2004). The summary of issues relating to prescribing of medicines by pharmacists and the contents of the proposed qualifications are presented below. Although no published papers were found that described findings of any evaluation of prescribing habits or practices of pharmacists in South Africa, 73.8% of graduates from the University of KwaZulu-Natal reported in a survey that they were adequately trained to provide pharmacy initiated therapy (Naidoo et al., 2009).

### 3.1 Conflict Between Dispensing Medical Doctors, Nurses and Prescribing Pharmacists

As far as 1998, Gilbert sounded the alarm as she wrote about ‘dispensing doctors and prescribing pharmacists’. In her papers she examined the issues and conflicts arising from the desire of pharmacists to extend their service offerings to include the right to prescribe medicines to patients; while medical doctors and dentists wished to engage in dispensing of medicines to their patients. Indeed, both medical doctors and pharmacists got what they wanted to some extent. Medical doctors were authorized by law to dispense medicines to their patients but could not and cannot operate a pharmacy. It took more years before they were required to undergo a ‘dispensing course’ for them to continue doing so. Pharmacists, on the other hand, were to take and obtain a Primary Care Drug Therapy qualification. With this qualification, in terms of Section 22A (15) of the Medicines and Related Substances Act 101 of 1965 of South Africa, they were allowed to diagnose and treat patients with a specified list of Schedule 3 and Schedule 4 medicines. As the need and the pressure to expand the delivery of health services increased, nurses were also required to do undergo a dispensing course so that they could dispense medicines at primary health clinics (Gilbert, 2001, 2004; National Department of Health, 2013a-c; Gray & Strasser, 1999; Gray & Suleman, 2012).

### 3.2 Authorized Pharmacist Prescriber Qualification

The advent of the new qualification will ensure that pharmacists are now going to get what they desired for so long. A postgraduate diploma in Pharmacy for Authorised Pharmacist Prescriber has been proposed and developed by the South African Pharmacy Council in order to meet the requirements of the National Human Resources Strategy for Health. This strategy calls specifically on the need to expand and improve service delivery in the provision of prescribing services to the people of South Africa. This qualification is aimed at extending the core clinical and pharmaceutical knowledge and skills acquired during undergraduate training and endeavor to develop skills of diagnosis and treatment so that pharmacists can treat patients with medicines listed in the Primary Health Care Essential Medicines List according to Primary Health Care Standard Treatment Guidelines of South Africa (National Department of Health, 2008, 2012; South African Pharmacy Council, 2011).

The ultimate purpose of extending prescribing to pharmacists is to increase access to health care services but this will also help to make better use of patient care skills of pharmacists that have been underutilized. It is known that by the time pharmacists complete their undergraduate degree, they have accumulated a lot of clinical skills. A look at their training programs shows that there is a substantial emphasis on clinical skills training. At Medunsa School of Pharmacy for instance, during the third and final year of training, almost half of notional hours and experiential or work integrated learning is devoted to clinical skills. Similarly, at the Nelson Mandela Metropolitan University School of Pharmacy, more than half of notional hours in the third year are devoted to clinical pharmacy (Gray & Strasser, 1999; Gray & Suleman, 2012; Summers et al., 2004; Nelson Mandela Metropolitan University, 2012; Medunsa School of Pharmacy, 2013).

For the proposed postgraduate degree, the exit level competencies expected are that the pharmacists who complete the training will be able to:

- Demonstrate clinical and pharmaceutical knowledge to diagnose and treat conditions commonly encountered in primary health care settings;
- Ensure quality prescribing practice;
- Perform clinical assessment to make a diagnosis;
- Formulate a treatment management plan;
- Implement treatment plan and monitor therapeutic outcomes;
- Evaluate the patient assessment and management processes of selected cases to ensure quality prescribing practice

The training is expected to last for 12 months including integrated assessments and a *practicum* period to be supervised by a designated clinical mentor. A variety of learning activities are proposed to be used in the delivery of this curriculum. These will include but not limited to:

- Class or lecture attendance for the theoretical concepts and tutorials
- Independent reading and studying by learners while being supported by academic staff
- Role-plays on some aspects as a preparation for real-world experiences
- Oral presentation by learners mainly presentation of cases
- Participation in group work mainly when preparing group assignments and role-plays
- Completion of observation forms and reflective reports during role-plays and practicum
- Design and development of portfolios of cases during the *practicum*

It is expected that this training will provide pharmacists with additional skills in clinical problem solving and their ability to communicate effectively with other health care professionals about patient care and management (South African Pharmacy Council, 2011; Gray & Suleman, 2012; Summers et al., 2004; Jones et al., 2005).

## 4. Discussion

The introduction of the authorized prescriber qualification will lead to some implications and challenges for community pharmacy practice as detailed below. The following discussion is not expected to be comprehensive but illustrative of issues that may arise.

### 4.1 Implications for Pharmacy Practice

#### 4.1.1 Conducting Diagnostics and Prescribing Treatment

Laureates of the new qualification will essentially have the right to examine, diagnose, prescribe and monitor the treatment of their clients or patients according to Primary Health Care Standard Treatment Guidelines of South Africa. They will be required to refer cases, that are either difficult for them or that are apparently falling within the realms of medical specialties, to relevant clinical specialists. To do so, they will have to conduct a formal consultation with the patient starting with history taking, through physical examination as well as ordering, or conducting and interpreting relevant diagnostic and laboratory tests (Naidoo et al., 2009; Gray & Suleman, 2012; Christensen & Farris, 2006).

#### 4.1.2 New Meaning of Pharmaceutical Care

In doing so, the concept of pharmaceutical care will take some new meaning in community pharmacies. Traditionally, pharmaceutical care has been the responsible provision of drug therapy for the purpose of achieving the elimination or reduction of a patient’s symptomatology; arresting or slowing of a disease process; or preventing a disease in close collaboration of the medical doctor and the patient in order to gain a correct understanding of the relevance and impact of the various medications on the patient’s pathology. In this new scenario, the authorized pharmacist prescriber will have to rely on him/herself, not necessarily in close collaboration with a medical doctor as this will be an independent prescribing model (MacLeod-Glover, 2011; Foppe van Mil & Fernandez-Llimos, 2013). For this reason, academic institutions offering this qualification would need incentives and encouragement to innovate and develop their training materials as to ensure that their teaching and assessments prepare candidates to be confident enough to be self-reliant. It is hoped that this will also give pharmacists the opportunity to conduct in-depth medication reviews, record and report adverse reactions (Malangu, 2006; Anderson et al., 2008).

#### 4.1.3 Required New Infrastructure

New infrastructure will be required for the expanded roles. These include the required equipment for conducting physical examination of patients, conducting rapid laboratory tests, and relevant paperwork or software for the ordering of laboratory assessment tests for diagnosis purposes. Similarly, reference books and manuals as well as facilities for patients’ waiting and consulting rooms may have to be expanded depending on the level of patronage in line with the Good Pharmacy Guidelines of South Africa (South African Pharmacy Council, 2010; South African Pharmacy Council- Pharmacy Act No. 53 of 1974).

#### 4.1.4 New Modalities of Interaction With Medical Aid Schemes

Customarily pharmacists used dispensing software programs to process their claims to medical aid schemes. With the expansion of their services, either the current software programs have to be upgraded to include codes for examination, diagnosis, and laboratory tests or additional software should be purchased for the purpose. This also implies that relevant induction and in-service training for both pharmacists and their support staff will be required on the use of the new tools. Therefore, pharmacists planning to undergo the postgraduate training to become authorized prescribers ought to pay attention to the training needs of their support staff members particularly those dealing with claims so that they are skilled enough to assist them with this important task (Sardinha, 1997; Malangu, 2006; Council for Medical Schemes, 2010).

#### 4.1.5 New Contribution in Occupational Health

Where the non-pharmacological treatment modality calls for a bed rest, the authorized pharmacist prescriber will have to recommend such for their qualifying patients. Consequently they will have to provide them with the relevant sick note or medical certificate. Employers would have to accept such certificates from their patients wishing to prove their incapacity to work. This is in line with the provision of Section 23 of the Basic Conditions of Employment as it states that “The medical certificate must be issued and signed by a medical practitioner or any other person who is certified to diagnose and treat patients and who is registered with a professional council established by an Act of Parliament”. Hence, sick notes issued by authorized pharmacist prescribers will be useful for occupational health purposes. However, it seems that authorized pharmacist prescriber will not be able to provide medical surveillance services because the Occupational Health and Safety Act of 1993 still stipulates that this function must be rendered by “an occupational medicine practitioner or a person who holds a qualification in occupational health recognized as such by the South African Medical and Dental Council as referred to in the Medical, Dental and Supplementary Health Service Professions Act, 1974 (Act No. 56 of 1974), or the South African Nursing Council as referred to in the Nursing Act, 1978 (Act No. 50 of 1978)”. Given the stated objectives of this qualification, there is a need for an amendment to this Act (Department of Labour- Basic Conditions of Employment, 2013; Mbatha et al., 2012).

#### 4.1.6 Clinical Trial Relating to Patient Care

Because of the fact that pharmacists were unable to diagnose and prescribe medicines, they could not act as principal investigators on clinical trials where a treatment decision directly affecting patient care had to be made. The advent of this new qualification opens a door for holders of this qualification to participate in drug development research for medicines and health conditions listed in the Primary Health Care Manual. It is hoped that this opportunity will be used fully by those interested in clinical research (Wright, 2010; National Department of Health, 2013e).

#### 4.1.7 Special Register for Authorized Pharmacist Prescribers

The proposed qualification will be of great interest to pharmacists in community pharmacy practice or those planning to practice in this area of pharmacy practice. Consequently, a special register for the laureates of this qualification as ‘specialist pharmacists” will have to be maintained by the South African Pharmacy Council (Gray & Suleman, 2012; South African Pharmacy Council, 2013).

#### 4.1.8 Expanded Areas for Continuing Professional Development

Depending of the local burden of diseases and the majority of the *strata* of the population that patronize a particular pharmacy, the authorized pharmacist prescriber would need to tailor his/her continuing professional development activities to include learning about new developments in the diagnosis, monitoring and treatment modalities of the diseases most frequently encountered within his/her geographical or catchment area. It goes without saying that some specific requirements might be required for the recognition of continuing professional development activities reported by authorized pharmacist’s prescribers (South African Pharmacy Council.-Continuing Professional Development, 2013; Grobbelaar, 2012).

### 4.2 Expected Challenges With Expanded Prescribing Responsibilities

#### 4.2.1 Risk of Undetected Prescribing and Dispensing Errors

Medications errors particularly prescribing errors constitute one of major challenges of modern health care service provision. Data from a systematic review show that prescribing errors occurred in 7% of medication orders and 50% of hospital admissions. Incorrect dosing has been the most common error (Lewis et al., 2009).

Recent studies from UK reported prescribing errors ranging from one in 8 patients to 43.8%. Similarly, dispensing errors have been reported to range from 0.01 to 3.32% in community pharmacies and 0.02 to 2.7% in hospital pharmacies (Avery et al., 2012; Seden et al., 2013; James et al., 2009).

In South Africa, Malangu and Nchabeleng reported that the prevalence of prescription errors was 27.1% in a cohort of patients on antiretroviral treatment and that though the majority of these errors were apprehended by pharmacists and corrected, 1.3% of these errors reached the patients and resulted in some temporary harm that necessitated hospitalization of the victims (Malangu & Nchabeleng, 2012).

The above findings are in the context where prescribing and dispensing activities have been performed by two different persons. With the change brought by the new qualification, in instances where the same person may perform the two functions, the possibilities of errors going undetected may be great (Malangu & Karamagi, 2010; James et al., 2011).

#### 4.2.2 Difficulty in Adjudicating Responsibility for Patient Harm

A related challenge is that even when two pharmacists are on duty at the same time so that they can check each other prescriptions, which would bear the biggest responsibility, should an error reach and harm a patient? It is therefore important that malpractice insurance should be considered by community pharmacies as an essential risk management strategy in this regard (Aronson, 2009; Coetzee & Carstens, 2011; Velo & Minuz, 2009; Anto et al., 2011).

#### 4.2.3 Confusion Among the General Public

In South Africa, consultation and physical examination have been the domains of medical doctors and nurses. For most patients, going to the ‘doctor’ meant preparing ones history and being ready to be examined. Hence in the eyes of the public, particularly the less educated one, a pharmacist consulting a patient will now be essentially a ‘doctor’. This situation is not entirely new as several countries in Africa, namely in Cote d’Ivoire, Kenya and Uganda, when pharmacy students complete their undergraduate studies and graduate they are referred to as “doctors” (Kenya Pharmacy & Poisons Board-Registration and Enrollment, 2013; Adunlin, 2013; Ministry of Health of Uganda- Pharmacy Council, 2013).

Since this new postgraduate qualification adds another year of training to the four year bachelor degree in pharmacy, it seems that curricular reforms leading to the holders of this qualification to be referred by the honorific title ‘doctor’ may be needed to allay the confusion and go ahead with the new trend that is already practiced in several other advanced economies such as France and the United States, where pharmacy students complete 6 years of studies and graduate as ‘Doctor of Pharmacy” (Gray & Suleman, 2012; Bourdon, 2008; Anderson & Futter, 2009).

### 4.3 Concluding Remarks

In conclusion, the new authorized pharmacist prescriber qualification will definitely expand the responsibilities of pharmacists and diversify their income streams from examining, diagnosing and monitoring treatment outcomes of their clients or patients. Accordingly, it augurs a new era for community pharmacy practice in South Africa. Moreover, the qualification brings in several implications and challenges that need to be managed. The pharmacy profession, academia, legislators and political decision-makers need to work together to resolve outstanding issues in a constructive manner. Overall, the positive aspects of this qualification seem to outweigh the challenges identified.
